# Digitalization and organizational capabilities: a mechanism-based integrative review

**DOI:** 10.3389/fpsyg.2026.1745412

**Published:** 2026-04-30

**Authors:** Sen Jia, Khai Wah Khaw, Yiqi Zhou

**Affiliations:** 1 School of Management, University of Science Malaysia, George Town, Malaysia; 2 Institut Asia-Europe, Universiti Malaya, Federal Territory of Kuala Lumpur, Malaysia

**Keywords:** digitalization, dynamic capabilities, mechanisms, organizational capabilities, systematic literature review

## Abstract

**Purpose:**

This study addresses the fragmented understanding of how digitalization shapes organizational capabilities by identifying and synthesizing the underlying mechanisms.

**Methods:**

A mechanism-based systematic literature review was conducted on 116 core studies published in Q1 journals between 2015 and 2024. A structured multi-stage screening and classification process was applied to identify key mechanisms and their interrelationships.

**Results:**

The findings reveal four mechanism families—enabling, cognitive, collaborative, and adaptive—and twelve sub-mechanisms explaining how digital structures, cognition, and collaboration interact to reconfigure organizational capabilities. Three dominant causal chains—enabling → adaptive, cognitive → adaptive, and collaborative → adaptive—are identified. Based on these results, the Digital Capability System (DCS) is proposed, conceptualizing capability evolution as a recursive, multi-level, and boundary-spanning system.

**Discussion:**

This study contributes by moving beyond linear antecedent–outcome models and advancing a mechanism-based, multi-level perspective on digital capability development. It also identifies key research gaps, including static treatments of infrastructure, output-biased innovation measures, and cross-level inconsistencies, and provides an agenda for future research and practical guidance for designing digitally enabled capability systems.

## Introduction

Digitalization has become a fundamental driver of the development and evolution of organizational capabilities (Teece, 2007; Vial, 2019). This is partly because the outcomes of digitalization do not hinge on the deployment of a single technology, but rather on top managers' systemic choices in strategic cognition, resource orchestration, and organizational design (Bharadwaj et al., 2013; Yeow et al., 2018). Consequently, a growing body of research has examined how digitalization shapes organizational adaptation and capability reconfiguration through enabling mechanisms (e.g., Nambisan et al., 2017), cognitive mechanisms (e.g., Leonardi, 2021; Matarazzo et al., 2021), and collaborative mechanisms (e.g., Björkdahl, 2020; Warner and Wäger, 2019). With the strategic importance of digitalization rising and organizational capabilities playing a decisive role in firm performance (Winter, 2003; Heredia et al., 2022), related research has surged over the past 5 years (Hanelt et al., 2021).

Existing reviews on digitalization and organizational capabilities mainly focus on the antecedents, processes, and outcomes of digital transformation (Hanelt et al., 2021; Verhoef et al., 2021), or provided a systematic overviews of its definitions, drivers, and performance effects (Vial, 2019; Saragih et al., 2024). Recent systematic reviews have also examined digital technology adoption and related organizational phenomena, highlighting the growing use of systematic literature reviews in digital research (e.g., Al-Sharafi et al., 2025). While these studies have yielded valuable insights into the phenomenon of digitalization, there remains, to the best of our knowledge, a lack of systematic integration and theorization of how digitalization shapes organizational capabilities through enabling, cognitive, and collaborative mechanisms. Focusing on these three mechanisms provides a useful entry point for understanding how digitalization reshapes organizational capabilities. Enabling mechanisms capture the technological and infrastructural foundations that support digital operations and information flows within organizations (Yoo et al., 2010; Bharadwaj et al., 2013). Cognitive mechanisms emphasize how digital technologies influence organizational learning, knowledge processing, and managerial sense-making (Leonardi, 2021; Nambisan et al., 2017). Collaborative mechanisms highlight how digital platforms and communication technologies reshape coordination patterns and knowledge exchange across organizational actors (Tiwana et al., 2010; Constantinides et al., 2018).

Compared with existing studies that primarily examine the antecedents or outcomes of digital transformation (Hanelt et al., 2021; Verhoef et al., 2021), this mechanism-based perspective helps uncover the underlying processes through which digitalization translates into capability development. By identifying the enabling conditions, cognitive processes, and collaborative interactions involved in digital transformation, this approach complements the existing literature and provides deeper theoretical insights into how organizational capabilities evolve in digital contexts (Vial, 2019; Nambisan et al., 2019).

We argue that the fundamental purpose of science is to advance theory, and theory, at its core, is a systematic explanation of causal relationships that requires clearly defined constructs, transparent causal chains, and theoretical mechanisms that reveal the logic linking variables (Bacharach, 1989). Theorizing mechanisms is particularly critical in this context, as it clarifies why and how digitalization influences organizational capabilities by uncovering the underlying processes, behaviors, and interactions, thereby enhancing both explanatory power and practical applicability (Merton, 1968; Thatcher and Fisher, 2021). Against this backdrop, this study conducts a mechanism-based systematic review of the digitalization–adaption mechanisms–organizational capabilities chain, with the aim of advancing cumulative, progressive, and practice-relevant scholarship in this domain.

We focus on enabling, cognitive and collaborative mechanisms as key points for understanding how digitalization shapes organizational capabilities. First, enabling mechanisms provide structural support through digital infrastructure, technological innovation and knowledge collaboration, which enhance organizational adaptability and innovation capacity (Bharadwaj et al., 2013; Nambisan et al., 2017). Second, cognitive mechanisms explain how organizations interpret digital signals through sensing, reflection and strategic transformation, so guiding capability reconfiguration (Leonardi, 2021; Matarazzo et al., 2021). Third, collaborative mechanisms highlight the role of internal and ecosystem-level collaboration in integrating distributed knowledge and resources, which supports capability development in digital environments (Warner and Wäger, 2019; Björkdahl, 2020). By systematically examining these three mechanisms, this study aims to clarify the causal pathways through which digitalization influences the evolution of organizational capabilities.

Our review systematically differs in scope and emphasis from other reviews on the broader digital transformation literature (Vial, 2019; Annarelli et al., 2021; Hanelt et al., 2021; Verhoef et al., 2021; Feliciano-Cestero et al., 2023; Saragih et al., 2024; Grego et al., 2024), with a particular focus on how digitalization shapes organizational capabilities through enabling, cognition, and collaboration mechanisms (see Supplementary Table A1 for a detailed comparison with existing reviews). In addition, compared with prior digital transformation reviews, our study is more systematic, selective, and oriented toward the construction of mechanism-based logic (see Table 1 for comparison). Unlike those reviews that mainly examine the antecedents and outcomes of digital transformation (e.g., Hanelt et al., 2021) or focus on conceptual definitions and performance implications (e.g., Vial, 2019; Verhoef et al., 2021), our review covers core studies published between 2015 and 2024 and systematically identifies and evaluates four categories of mechanisms and twelve sub-mechanisms. To our knowledge, this is among the first systematic reviews to conceptualize “mechanism chains” as central, examining how digitalization shapes organizational capabilities. Methodologically, we restricted our sample to SCI/SSCI Q1 journals and applied citation thresholds together with repeated manual screening to ensure that the included articles are both academically influential and highly relevant to the research topic. Unlike prior reviews with broader scope but inconsistent standards (e.g., Annarelli et al., 2021; Saragih et al., 2024; Grego et al., 2024), our study ensures greater rigor and representativeness.

**Table 1:** Comparison with existing reviews on digitalization.

	Our review	(Hanelt et al. 2021)	(Verhoef et al. 2021)	(Vial 2019)
Review topic	How digitalization shapes organizational capabilities: a mechanism-based systematic review and future research agenda	A systematic review of digital transformation literature: insights and implications for strategy and organizational change	Digital transformation: multidisciplinary reflections and a research agenda	A systematic literature review on digital transformation: insights and implications for strategy and organizational change
Period	2015–2024	2000–2018	2000–2020	2000–2019
Journal	51 SCI/SSCI Q1 Journals	23 management and information systems journals	27 journals in management, information systems, and marketing	Management, information systems, strategy journals, and interdisciplinary outlets
Field(s)	Management, strategy, information systems, and entrepreneurship	Management and information systems	Management, marketing, and information systems	Management, strategy, and information systems
Database(s)	WoS, Google Scholar	WoS, Scopus	WoS, Scopus	WoS, Scopus, Google Scholar
Number of articles	Initially identified 1,177 → screened 584 → 116 core studies	Initially identified 1,800 → 93	Initially identified 1,420 → 94	Initially identified 282 → all 282 retained

Our review systematically differs from other reviews on the broader digital transformation literature (Vial, 2019; Hanelt et al., 2021; Verhoef et al., 2021), which primarily center on the antecedents, processes, and outcomes of digital transformation, or synthesize its definitions, drivers, and performance implications (see Supplementary Table A1 for a detailed comparison with existing reviews). In addition, compared with extant reviews on digital capabilities and related domains (e.g., Annarelli et al., 2021; Grego et al., 2024; Feliciano-Cestero et al., 2023), our review is also more systematic, selective, and emphasizes the integration of mechanisms at the management and strategic levels (see Table 1 for comparison). Unlike those reviews with narrower topical foci—such as (Annarelli et al. 2021), which concentrates on the antecedents and outcomes of digital capabilities; (Grego et al. 2024), which traces the conceptual evolution of IT capabilities and digital capabilities; or (Feliciano-Cestero et al. 2023), which highlights the role of international contexts in shaping digitalization effects—our review focuses on how digitalization shapes organizational capabilities through enabling, cognition, and collaboration mechanisms. We further identify adaptation mechanisms as a fourth mechanism family, which capture the explanatory processes through which digitalization is translated into capability development. Accordingly, all four categories are conceptualized as theoretical mechanisms rather than empirical mediators. This approach offers a more comprehensive and mechanism-oriented examination of this topic to date.

Building on this synthesis, we conceptualize the Digital Capability System (DCS), a novel theoretical framework that reconceptualizes how digitalization shapes organizational capabilities. While prior reviews have primarily depicted linear chains between antecedents and outcomes (Vial, 2019; Verhoef et al., 2021; Hanelt et al., 2021), we advance a system perspective that emphasizes recursive feedback, cross-mechanism complementarities, and multi-level contingencies. The DCS integrates enabling, cognitive, collaborative, and adaptation mechanisms into a dynamic constellation that jointly drives capability evolution. Importantly, we conceptualize these mechanisms as theoretical explanatory processes rather than empirical mediators. This conceptualization aligns with the call for reviews to contribute to theory building rather than mere aggregation (Bacharach, 1989; Cronin and George, 2023), and it positions our work not only as a synthesis but also as the basis for theorizing a recursive and multi-path capability system.

Building on this conceptualization, this study aims to the following research question: How does digitalization shape the development and reconfiguration of organizational capabilities, and through which mechanisms does this process unfold?

## Methods

### Sample and data

**Figure 1:**
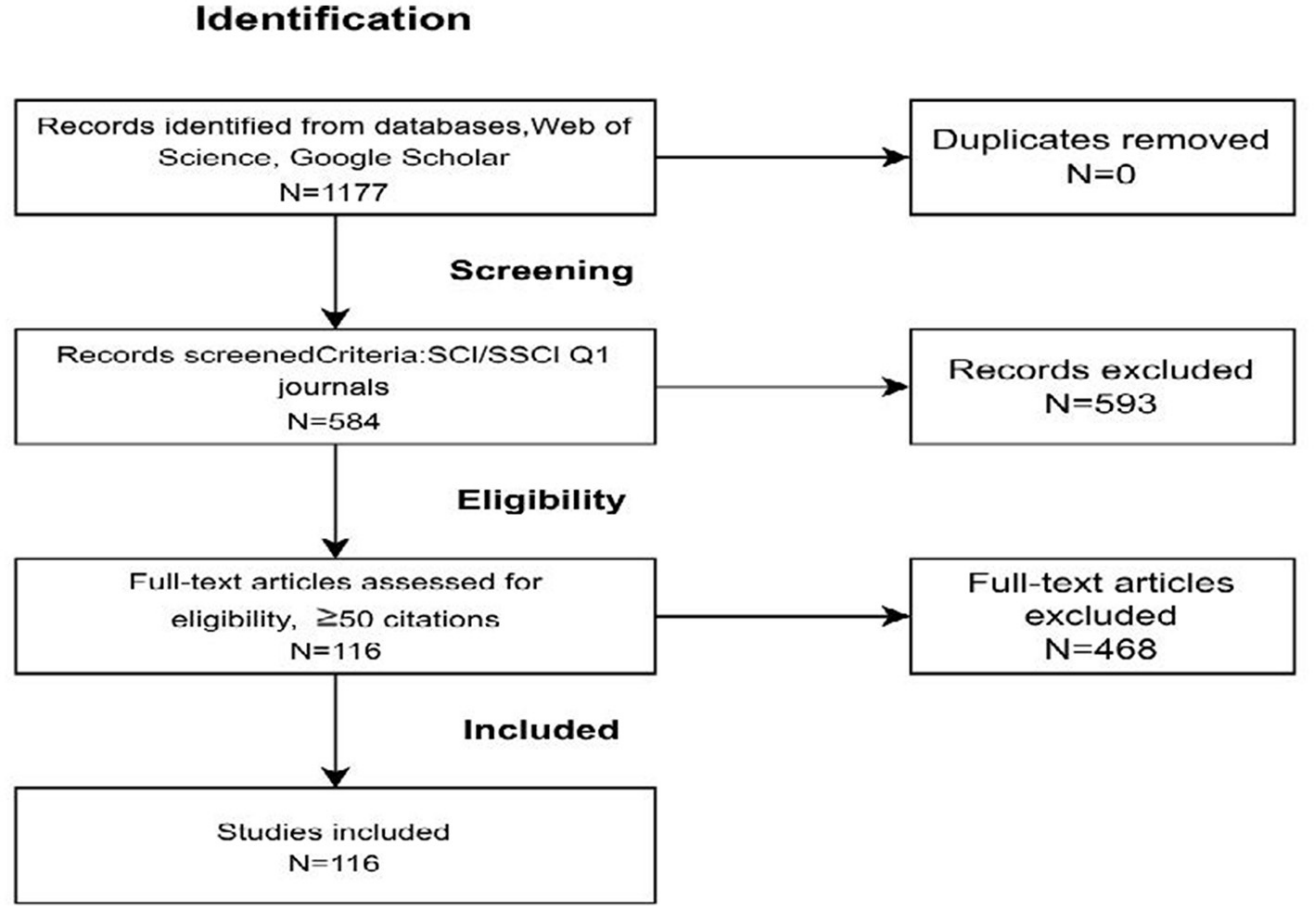
PRISMA 2020 flow diagram of the study selection process.

Following recent recommendations for sample selection in systematic literature reviews within management research (e.g., Kraus et al., 2020; Mohamed Shaffril et al., 2021; Hiebl, 2021; Simsek et al., 2023; Varsha et al., 2024), the review process was guided by the research question introduced in the Introduction: “*How and through which mechanisms does digitalization influence organizational capabilities?”* To operationalize this question within a systematic literature review, we adopt the PICOS framework on define the scope of the review and guide the literature research, screening and coding procedures in the methods section (as shown in Figure 1, Table 2).

**Table 2:** PICOS framework for the systematic literature review.

PICOS element	Description in this study
Population	Organizations and firms undergoing digitalization or digital transformation
Intervention	Digitalization-related structures and practices (e.g., digital infrastructure, digital governance, data platforms, AI technologies)
Comparison	Different organizational contexts, industries, or digital maturity levels
Outcomes	Organizational capabilities such as innovation capability, resource integration, organizational learning, and exploration–exploitation balance
Study design	Empirical and conceptual studies published in peer-reviewed SCI/SSCI journals

Before constructing the search strategy, we first clarified the operational definitions of the two core concepts. Regarding digitalization, following Vial's (2019) review, digitalization is defined as a process that triggers systemic changes in an organization's strategy, processes, and structures through the introduction and integration of digital technologies. This process involves not only the deployment of technological resources (e.g., digital infrastructure, data platforms) but also the redesign of business models and operational practices (Hanisch et al., 2023). Moreover, (Yoo et al. 2010) emphasize that digitalization reshapes organizational boundaries through modular and platform logics, highlighting that it represents not merely a technological upgrade but a fundamental reconfiguration of organizational functioning mechanisms. With respect to organizational capabilities, we adopt Winter's (2003) definition, which views them as repeatable and reliable patterns of action required for performing organizational tasks. This definition underscores that capabilities differ from individual resources and final performance outcomes, functioning instead as the processual link between the two. Typical manifestations of organizational capabilities include innovation capability, resource integration, organizational learning, and the balance between exploration and exploitation (Amit and Schoemaker, 1993). By contrast, dynamic capabilities (Teece et al., 1997) and absorptive capacity (Zahra and George, 2002) are better conceptualized as adaptation-related mechanisms that explain how digitalization translates into organizational capabilities, rather than as ultimate dependent variables. In some empirical studies, these constructs are operationalized as mediators in statistical models, but in this review they are treated at the theoretical level as mechanisms.

Building on this foundation, we refined and expanded the initial keyword set by drawing on both foundational empirical studies (e.g., March, 1991; Teece et al., 1997; Sirmon et al., 2007; Verhoef et al., 2021) and recent systematic reviews (e.g., Tilson et al., 2010; Constantinides et al., 2018; Yeung, 2017; Abraham et al., 2019; Vial, 2019; Hanisch et al., 2023). The final search list comprised two categories: (1) digitalization-related keywords—digital governance, digital infrastructure, digital transformation, platform governance, data governance, and algorithmic governance; and (2) organizational-capability-related keywords—organizational capability, dynamic capabilities, absorptive capacity, organizational learning, resource integration, innovation capability, exploration, and exploitation. Although constructs such as dynamic capabilities and absorptive capacity are sometimes discussed as forms of organizational capabilities in the literature, in this review they are conceptually interpreted as adaptation-related mechanisms that explain how organizations integrate and reconfigure digital resources in response to technological change. In some empirical studies, these constructs are operationalized as mediators, but here we treat them at the theoretical level as mechanisms. These constructs were therefore included as search keywords to ensure comprehensive literature coverage, while their theoretical role is interpreted as mechanisms linking digitalization and capability development in the subsequent analysis.

The search strings combined digitalization-related terms with organizational capability-related terms using Boolean operators (AND/OR). For example (“digital transformation” OR “digitalization” OR “digital infrastructure”) AND (“organizational capability” OR “dynamic capability” OR “absorptive capacity”). The literature search was conducted primarily using the Web of Science (WoS) database, covering publications from 1949 to 2025. Google Scholar was used as a supplementary tool for citation tracing and cross-checking influential studies, ensuring that highly cited publications were not overlooked. The detailed search strategy used in the Web of Science database is reported in Appendix A. Detailed definitions and sources are shown in Table 3.

**Table 3:** Definition of search terms.

Search	Definition
Digital governance	The mechanism of allocating decision-making authority and delineating responsibilities regarding digital resources within the organization (Hanisch et al., 2023)
Digital infrastructure	Reconfigurable and scalable IT resources and platform components (Tilson et al., 2010)
Digital transformation	A strategic, cross-level process of organizational transformation (Vial, 2019)
Platform governance	The interactive governance mechanism among users, technologies, and rules within a platform (Constantinides et al., 2018)
Data governance	Standards for data quality control, privacy, security, and utilization (Abraham et al., 2019)
Algorithmic governance	Process automation and management through algorithmic rules and models (Yeung, 2017)
Organizational capability	The overall set of capabilities required for an organization to perform tasks (Winter, 2003)
Dynamic capabilities	A tri-dimensional framework of sensing, seizing, and transforming capabilities (Teece et al., 1997)
Absorptive capacity	The ability to absorb, transform, and apply new knowledge (Zahra and George, 2002)
Organizational learning	Mechanisms through which organizations accumulate and refine practices based on experience (Crossan et al., 1999)
Resource integration	The capability to orchestrate and deploy heterogeneous resources in combination (Sirmon et al., 2007)
Exploration and exploitation	The ability to balance innovation and stability (March, 1991)

During the retrieval and screening process, we initially identified 1,177 candidate articles through Web of Science (WoS) database, with Google Scholar used as a supplementary tool for citation tracing and cross-checking influential studies. We then narrowed the scope to SCI/SSCI Q1 journals via the WoS filtering function, while also including outlets listed in the FT50 and UTD24 rankings, thereby ensuring both academic authority and interdisciplinary representation.

After this step, 584 candidate papers remained. To further enhance relevance and focus the review on influential contributions, we applied an additional citation-based filter (≥50 citations). The citation distribution of the candidate articles (n = 584) was highly right-skewed (median = 12; mean = 46; 75th percentile = 37; max = 3,004), indicating a long-tail pattern in which a small subset of publications receives disproportionate scholarly attention. We therefore used ≥50 citations as an operational cutoff to capture the high-impact tail of the literature. This process yielded a final core sample of 116 articles. At the same time, we acknowledge that citation-based thresholds may disadvantage recently published studies that have not yet accumulated substantial citations. To mitigate this limitation, the citation filter was complemented with manual screening and relevance checks, allowing the inclusion of theoretically important and recently published studies even if their citation counts had not yet reached the threshold.

To minimize potential bias, the review focused on high-quality outlets indexed in SCI/SSCI Q1 journals and leading rankings such as FT50 AND UTD24. To provide a descriptive overview of the literature, we summarized the temporal distribution of the selected studies. The temporal distribution of the reviewed studies is presented in Table 4.

**Table 4:** Distribution of the reviewed studies.

Year	Number of articles	Representative journals
2015	1	Journal of management information systems
2016	0	–
2017	1	MIS quarterly
2018	1	Information system journal
2019	6	Business horizons
2020	17	British journal of management
2021	29	Journal of business research
2022	25	Technological forecasting and social change
2023	30	Journal of business research
2024	6	Annals of operations research
Total	116	

Representative journals include *Journal of Management Studies, Journal of International Business Studies, MIS Quarterly, Technological Forecasting and Social Change, Supply Chain Management: An International Journal*, and *Journal of Business Research*. These outlets are mainstream or high-impact journals in their respective fields and thus provide a comprehensive reflection of research progress on digitalization and organizational capabilities.

These journals are recognized as mainstream or high-impact outlets in their respective fields, providing a comprehensive reflection of research progress on digitalization and organizational capabilities. It should be noted that although the search period spanned 1949–2025, the final sample was almost entirely concentrated in 2015–2024 due to the rapid development of the research topic in the past decade and the natural convergence effect of the citation threshold. More than 70% of the selected studies were published within the last 5 years. The final sample includes 116 articles. Supplementary Table A2 provides a list of these articles, sorted by author names, year of publication, and source journal. Other columns in the table further report the specific independent variable (IV), the actor(s) (e.g., entire organization, specific department, or inter-organizational network), and the dependent variable (DV). The last columns display the digitalization dimension, the organizational capability dimension, the logic path, and the theoretical mechanism, and additionally indicate the mediating and moderating variables.

### Coding protocol

**Figure 2:**
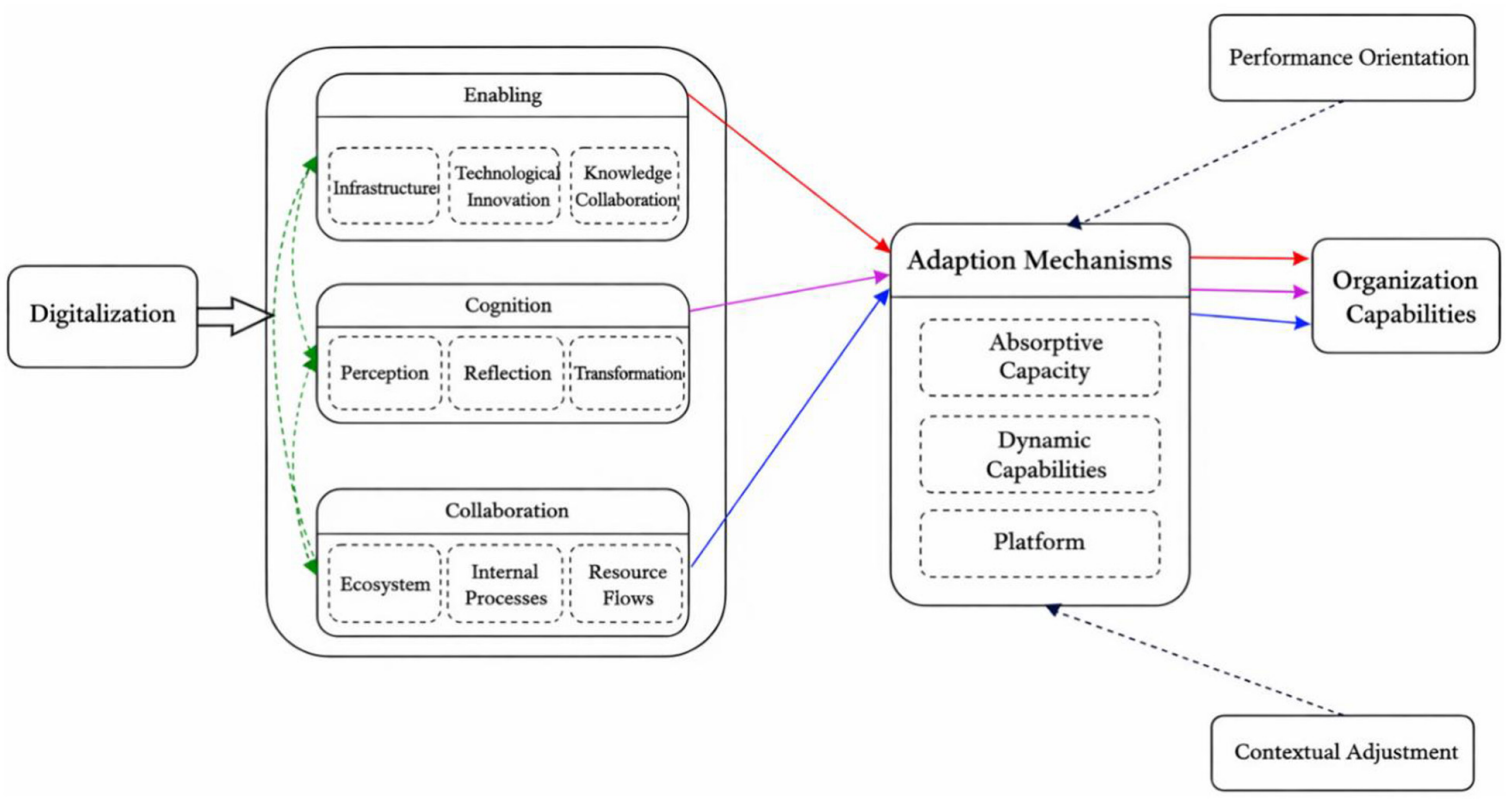
The digital capability system framework.

Following best practices for conducting rigorous and impactful literature reviews (e.g., Cronin and George, 2023; Simsek et al., 2023), we created columns in Excel to code the basic information of each article in the final sample, including (a) Key publication information (e.g., author, year, title, journal) (b) Dependent variable, independent variable, moderating variable and mediating variable, as well as key constructs and relationships. (c) Logical path, theoretical mechanism. Specifically, we first identified and summarized the constructs related to digitalization and organizational capabilities and coded the specific relationships examined. We then reviewed the theoretical arguments that generated the relevant hypotheses and propositions. Next, we identified the specific mechanisms underpinning the relationship between digitalization and organizational capabilities and summarized these mechanisms, which were subsequently classified according to their nature and content. To ensure inter-coder reliability and the accuracy of the review, at least two authors examined the mechanisms in each study and developed a consensus-based summary, and at least two authors also reached consensus in assigning each mechanism to a category; when divergent interpretations emerged, we revisited the full text, re-examined the theoretical arguments to resolve disagreements, and, when necessary, invited additional team members to participate in the discussion. We identified and defined four key mechanisms: enabling (infrastructure, technological innovation, knowledge collaboration), cognition (transformation, reflection, sensing), collaboration (ecosystems, internal processes, resource flows), and adaptation mechanisms (absorptive, dynamic, platform). We then examined how different mechanisms combine to explain the impact of digitalization on organizational capabilities and identified the causal pathways from digitalization to organizational capabilities. We also drew on commentary in the broader organizational capabilities literature (Warner and Wäger, 2019; Ghosh et al., 2022) to identify potential causal pathways for future research. This process resulted in an integrated framework, as illustrated in Figure 2, which we describe in detail below.

## An intergrative and synthesizing framework

Figure 2 highlights our mechanism-centered review framework, illustrating how digitalization structures influence organizational capabilities through four sets of mechanisms: enabling (infrastructure, technological innovation, knowledge collaboration), cognition (transformation, reflection, sensing), collaboration (ecosystems, internal processes, resource flows), and adaptation mechanisms (absorptive, dynamic, platform). Table 5 systematically organizes the main theoretical mechanisms and their underlying meanings in the digitalization–organizational capabilities literature, and for each mechanism specifies actionable measurement dimensions and indicators, thereby providing a unified reference framework for subsequent empirical research and comparative analysis.

**Table 5:** Digitalization and organizational capabilities: mechanisms and indicators.

Theoretical	Mechanism	Description	Measurement dimensions/variables
Enabling	Infrastructure	Underlying communication and computing systems shape the embedding of digitalization in business processes and its translation into organizational capabilities	Platform availability, network connectivity, data interoperability, IT investment, degree of external resource linkage, cross-level adaptability
	Technological innovation	Embedding digital technologies drives optimization, reconfiguration, and capability evolution across structural, cognitive, ecosystem, and sustainability dimensions	Number of patents, R&D expenditure, technology adoption rate, frequency of cross-departmental collaboration, extent of green technology deployment, platform participation level
	Knowledge collaboration	Actor interactions in knowledge sharing and co-creation accelerate sensing and response, driving capability reconfiguration	Number of collaborations, frequency of knowledge sharing, efficiency of cross-stage knowledge transformation, degree of cross-boundary platform participation, external knowledge absorption rate
Cognition	Perception	The ability to identify, filter, and interpret external information underpins the cognitive basis for coping with uncertainty	Frequency of environmental scanning, accuracy of signal recognition, proportion of data-driven decisions, cross-level perception consistency, information response time
	Reflection	Platforms and data feedback enable cognitive reconfiguration and opportunity/resource reallocation	Frequency of top management attention redirection, scope of information acquisition, rate of cognitive updating, extent of inter-organizational information sharing
	Transformation	Embedding cognition into models and processes institutionalizes technological gains and aligns strategy with structure	Technology embedding; process reconfiguration share; cross-departmental integration speed; degree of institutionalization; top-down and bottom-up coordination level
Collaboration	Internal processes	Structural and process optimization enhance resource orchestration and responsiveness, aligning data and capabilities across the value chain	Frequency of cross-departmental collaboration, index of process standardization and flexibility, speed of knowledge integration, proportion of internal data interoperability
	Ecosystems	A shared platform integrating internal knowledge with external data and resources enables cross-boundary co-creation and joint capability building	Number of ecosystem partners, platform openness, frequency of cross-boundary collaboration, proportion of resource sharing, diversity of governance structures
	Resource flows	Long-term resource interaction and adaptation enable adjustments to cooperation structures and business models under environmental change	Duration of alliances, frequency of resource exchanges, rate of cross-domain data sharing, number of cooperation adjustments
Adaptation	Absorptive	The ability to identify, assimilate, and transform external knowledge serves as a key mechanism linking digital enabling and innovation performance	Technology adoption rate, number of patents, frequency of collaborations, knowledge transformation rate, speed of cross-level knowledge diffusion
	Dynamic	Dynamic capabilities (sensing–seizing–reconfiguring) drive strategic–capability–cultural alignment and sustain resilience and agility	Speed of process adjustments, frequency of cross-departmental integration, proportion of project reconfiguration, response time to external shocks
	Platforms	Platform-based infrastructure integrating data and interfaces to enable cross-organizational sensing and resource reconfiguration	Number of platform connections, volume of cross-platform data flows, number of standardized interfaces, degree of external developer participation

The framework highlights three main explanatory patterns in the literature, marked by red, blue, and pink solid arrows. The first causal chain (red arrow) indicates that enabling mechanisms (e.g., infrastructure, technological innovation, knowledge collaboration) influence the development and evolution of organizational capabilities through adaptation related mechanisms, a process that relies on the deep embedding of digital resources in structures, processes, and responsibilities (Siachou et al., 2021; Wu et al., 2023). The second pattern (pink arrow) emphasizes that cognitive mechanisms (sensing, reflection, transformation) drive the enhancement of organizational capabilities through adaptation mechanisms, with the core lying in how digitalization-enabled information visualization and data feedback accelerate cognitive iteration (Feliciano-Cestero et al., 2023; Mele et al., 2024). The third pattern (blue arrow) illustrates how collaboration mechanisms (internal processes, ecosystems, resource flows) foster the reconfiguration of organizational capabilities through adaptation mechanisms, thereby promoting capability synergies across departments and organizations (Chen and Tian, 2022; Plekhanov et al., 2022; Sjödin et al., 2023).

The figure also includes green dashed arrows, which indicate explanatory linkages that are present in current research but remain underexplored, such as the interactions among enabling, cognition, and collaboration mechanisms, as well as the dynamic interlinkages across different dimensions of adaptation mechanisms. These relationships are often hypothesized in existing studies but lack systematic empirical examination.

In addition, the black dashed arrows in the framework represent potential directions for linking the mechanisms identified in this review with the broader organizational capabilities and digital transformation literature, such as incorporating mechanisms that have been less explored in external domains (e.g., slack resources, organizational resilience, resource reconfiguration) to enrich the theoretical model of digitalization–adaptation mechanisms–organizational capabilities. Such extensions not only help to fill gaps in the literature but also provide entry points for comparative studies across contexts and actors.

Our review not only systematically integrates the main theoretical mechanisms linking digitalization, adaptation mechanisms, and organizational capabilities, but also extends this framework by conducting a comparative analysis with the broader organizational capabilities and digital transformation literature. In doing so, we identify external mechanisms that have received little in-depth attention in current research, such as slack resources, organizational resilience, and resource reconfiguration. These mechanisms have been shown in strategic management and dynamic capabilities theory to be closely related to capability evolution (Eisenhardt and Martin, 2000; Sirmon et al., 2007), yet they remain insufficiently embedded in digitalization-driven capability formation pathways. Incorporating such external mechanisms into our framework (denoted by black dashed arrows) not only helps to bridge the gap between digital transformation research and the broader organizational capabilities literature but also provides entry points for comparative studies across contexts and actors. By comparing the integrative potential of different mechanisms and causal chains (Cronin and George, 2023) and drawing on the methodological logic of holistic triangulation (Turner et al., 2017), our framework highlights diverse pathways for future research to advance capability reconfiguration and sustainable competitive advantage in complex environments.

## Theoretical mechanisms and causal pathways

Our review reveals four groups of theoretical mechanisms, twelve sub-categories, and three main explanatory patterns linking digitalization to organizational capabilities. Building on these three patterns—(1) enabling–adaptation mechanisms–organizational capabilities, (2) cognition–adaptation mechanisms–organizational capabilities, and (3) collaboration–adaptation mechanisms–organizational capabilities—we structured the present review. We conducted a critical assessment of the literature and, as summarized in Table 6, identified opportunities for advancing research within each of these explanatory configurations. The section on future research opportunities further elaborates on ways to extend this body of work, with particular attention to opportunities related to the integration of different categories of mechanisms.

**Table 6:** Critical evaluation of major causal chains and mechanisms.

Causal chains	Mechanisms	Critical evaluation
Empowerment–adaptation mechanisms–organizational capabilities	Infrastructure	Digital infrastructure is widely regarded as the foundation of digital enablement, supporting communication, collaboration, and computational capacity (Omrani et al., 2022; Zhang Y. et al., 2023). However, some studies treat infrastructure as a static resource, overlooking post deployment adaptation and capability internalization (Annarelli et al., 2021). Empirical research also relies on indirect indicators such as platform availability conversion mechanism (Teubner and Stockhinger, 2020).
	Technological innovation	Most studies link technology adoption with capability enhancement (Warner and Wäger, 2019; Jafari-Sadeghi et al., 2021). However, they often overlook mediating mechanisms between innovation adoption and capability formation, leaving the transformation chain underexplored. Measurements mainly rely on output indicators such as patents and R&D investment, while process indicators and cross industry evidence remain limited (Ciampi et al., 2022; Wiesböck et al., 2020).
	Knowledge collaboration	Knowledge collaboration enhances organizational capabilities (Kodama, 2020; Li et al., 2022), but barriers (e.g., fragmentation, weak governance, misalignment) are often overlooked. Prior studies focus on collaboration intensity, with limited evidence on knowledge embedding and failure mechanisms across the acquisition–assimilation–application process (Scuotto et al., 2021; Jayashree et al., 2022).
Cognition–adaptation mechanisms–organizational capabilities	Perception	Sensing capability builds on the integration of external knowledge and digital technologies, with absorptive capacity linking signal recognition to capability reconfiguration (e.g., Trantopoulos et al., 2017; Volberda et al., 2021). Digital literacy and multi-level cognitive differences further shape transformation trajectories (e.g., Matarazzo et al., 2021; Dabrowska et al., 2022), however existing research remains largely static and context-specific, with limited evidence on cross-level mechanisms and temporal dynamics (e.g., Gfrerer et al., 2020).
	Reflection	Reflection is widely regarded as a key mechanism for strategic adjustment and capability reconfiguration, shaping executive attention, organizational reframing, and resource reallocation in digital contexts (Li et al., 2018; Zeng et al., 2019; Khan et al., 2021; Di Vaio et al., 2021; Feliciano-Cestero et al., 2023). However, studies diverge in how reflection is conceptualized and institutionally embedded. The predominance of context-specific case analyses limits systematic explanations of cross-level causal chains and institutional constraints.
	Transformation	Most studies attribute transformation to complementarities between absorptive capacity and innovation strategies, embedding cognition into business model redesign and process reconfiguration (Müller et al., 2020; Scuotto et al., 2022). Evidence spans strategic transformation, digital organization, search optimization, and internationalization (Jafari-Sadeghi et al., 2023; Malodia et al., 2023). However, the literature diverges between bottom-up and top-down pathways, while under-specifying meso-level coordination and feedback dynamics. Contextual constraints are often treated as exogenous, overlooking nonlinearity, feedback, and decay. As a result, the transformation–adaptation–capability link remains fragmented.
Collaboration–adaptation mechanisms–organizational capabilities	Internal processes	Digitalization is widely viewed as enhancing organizational responsiveness by reshaping structures, governance layers, and information flows, thereby improving cross-departmental coordination and process transparency (Klos et al., 2021; Ritala et al., 2021; Plekhanov et al., 2022). However, studies diverge in the logics of process transformation, emphasizing either platform-driven redesign or managerial-led reorganization. Cross-departmental feedback mechanisms and the linkage between internal process optimization and external collaboration remain insufficiently examined.
	Ecosystems	Open innovation platforms, strategic alliances, and cross-border networks enable firms to embed in ecosystems and achieve value co-creation (Crupi et al., 2020; Arias-Pérez et al., 2021). Empirical evidence spans cluster collaboration and global business models (Nayal et al., 2022; Meyer et al., 2023). However, the literature diverges on ecosystem governance (centralized vs. decentralized), while exit and reconfiguration mechanisms remain underexplored.
	Resource flows	Resource orchestration—particularly structuring, bundling, and leveraging—is widely viewed as a key mechanism for managing environmental uncertainty in digital transformation (Chen and Tian, 2022; Huang et al., 2022). Studies also highlight the integration of AI and dynamic capabilities in enabling new business model innovation (Sjödin et al., 2023). However, the literature diverges on whether resource dynamics are driven primarily by internal capability reconfiguration or external network relationships, while cross-industry and cross-stage evidence remains limited.
Adaptation mechanisms	Absorptive capacity	Absorptive capacity is widely recognized as a key mediator linking digital resources and organizational capabilities (e.g., Bordeleau et al., 2019; AlNuaimi et al., 2022; Chatterjee et al., 2023). However, studies diverge on its drivers, focusing either on external openness and inter-organizational linkages (e.g., Dabić et al., 2023) or internal R&D
		accumulation and skill development (e.g., Tortorella et al., 2020; Jiang et al., 2022). Empirical measures typically rely on proxies such as technology adoption or patents, while cross-level processes and temporal dynamics remain underexplored.
	Dynamic capabilities	Dynamic capabilities are commonly proxied by “speed–scale–agility” metrics, approximating sensing–seizing–reconfiguring via platform access, launch rates, or project counts (e.g., Singh et al., 2021; Ciampi et al., 2022). reconfiguration depends on post-deployment processes shaped by structural power and cross-level interpretation, and collaboration often remains superficial (e.g., Del Giudice et al., 2021; Khurana et al., 2022). Evidence is also concentrated in single industries and conflates levels of analysis, raising reverse-causality concerns and obscuring temporal and mediating roles (e.g., Karimi and Walter, 2015; Soluk and Kammerlander, 2021).
	Platforms	Platform-based integration and process collaboration are often assumed to enhance adaptive effectiveness (Hanelt et al., 2021; Skare et al., 2023). However, the link between collaboration, behavioral change, and capability reconfiguration involves multiple mediating processes, and technology adoption may be constrained by power structures, routines, and governance arrangements (Chen et al., 2022). Measurement typically relies on proxies (e.g., launch rates, cross-departmental traffic), with limited attention to platform governance, power asymmetries, and industry concentration, which may generate lock-in effects and survivor bias (Di Vaio et al., 2023).

### Enabling–adaptation mechanisms–organizational capabilities

Enabling is classically defined as the process of eliminating powerlessness and enhancing the sense of competence and control of individuals or groups, thereby increasing their effectiveness in achieving goals (Conger and Kanungo, 1988; Zimmerman, 1995). In organizational research, enabling mechanisms typically encompass four dimensions: structural support—accessibility of institutions and resources; psychological enabling —meaning, autonomy, and a sense of efficacy; relational interaction—supportive networks and collaborative mechanisms; dynamic adjustment—the ability to maintain goal orientation and behavioral flexibility during change (Spreitzer, 1995). In the digitalization context, the logic and boundaries of these mechanisms have undergone significant transformation: structural support is driven by digital infrastructure, dynamic adjustment is enabled by technological innovation, and relational interaction unfolds through knowledge collaboration (Yeow et al., 2018). Moreover, existing reviews highlight that digital enabling is not merely a simple aggregation of isolated mechanisms, but rather a cross-level, multi-path systemic process that shapes organizational capabilities through the dynamic coupling of resources, capabilities, and institutions (Nambisan et al., 2017). Accordingly, this study conceptualizes enabling mechanisms—often referred to in the literature as digital enabling, but here strictly understood as technological enabling processes—as the starting point of three mechanism family (“infrastructure–technological innovation–knowledge collaboration”) that drive the development and evolution of organizational capabilities.

Infrastructure can be defined as the communication, collaboration, and computing systems that support digital technologies (Tilson et al., 2010; Henfridsson and Bygstad, 2013). It includes not only the physical layer of hardware and networks, but also the logical structure of data, platforms, and integration capabilities (Nambisan et al., 2017; Ancillai et al., 2023). Within the framework of digital enabling, infrastructure serves not only as a prerequisite for technology deployment but also as the structural foundation for efficient information flow, rapid data processing, and system interoperability (Vial, 2019). A robust infrastructure facilitates the embedding of digital technologies into business processes, strengthens cross-departmental collaboration, and enhances resource integration (Yeow et al., 2018). However, in practice, many organizations face infrastructure deficiencies or lack proper alignment, which hinders the effective integration of digitalization into daily operations and thereby weakens the enabling effects of technology (Wiesböck and Hess, 2019; Sousa-Zomer et al., 2020). The completeness and reconfigurability of digital infrastructure constitute essential conditions for digitalization to foster organizational sensing, collaboration, and adaptive capacity (Kong and Liu, 2023).

Within the infrastructure enabling mechanism, it is the degree of readiness and embeddedness that determines whether digital resources can be transformed into organizational capabilities (Vial, 2019). Prior studies note that flexible and reconfigurable IT infrastructures, by adapting to changes in business processes under dynamic environments, support both digitalization and the reconfiguration of organizational capabilities (Yeow et al., 2018; Zeng et al., 2019). From different theoretical perspectives, this role has been validated: (Wu et al. 2023), for instance, points to structural support that fosters process integration and collaboration, whereas (Karimi and Walter 2015) stress its role in facilitating integration–adaptation processes. Deep coordination between technological and organizational elements is therefore viewed as a key condition for turning digitalization into organizational capabilities (Song, 2019; Bag et al., 2020). Case-based research further indicates that digital technologies only drive capability development when embedded into business processes in close alignment with organizational structures and IT architectures (Wiesböck and Hess, 2019). IT infrastructure, as (Vial 2019) notes, improves information fluidity and coordination efficiency, which in turn creates favorable conditions for process integration and the development of dynamic capabilities. According to (Li et al. 2018), digital infrastructure proves effective only when technology and organization adapt to each other, enabling interoperability and resource reconfiguration.

External linkages play a critical role in fostering capability integration and transformation (North et al., 2019). Evidence from city-level large-sample data shows that infrastructure connectivity facilitates regional knowledge flows and technological diffusion, while at the same time strengthening firms' structural sensitivity and digital absorptive capacity (Zeng et al., 2019). (Wu et al. 2023) demonstrate that access to and integration of external digital resources shape network embeddedness, thereby supporting renewal and system-wide cooperation. Using a natural experiment, (Chen et al. 2023) further reveal that fiscal external resources enhance firms' adoption of digital services and technologies, which indirectly contributes to the development of internal capabilities.

Technological innovation is embedding digital technologies into processes and structures to enhance performance, marked by embeddedness, combinability, and complementarity (Jafari-Sadeghi et al., 2021; Babu et al., 2021; Fang and Liu, 2024). As the core engine of digital enabling, it activates organizational sensing, responsiveness, and integration capabilities by driving process reengineering, breaking organizational boundaries, and reshaping decision-making mechanisms (Akter et al., 2020). Studies have shown that the deployment of technologies such as big data, artificial intelligence, and blockchain can significantly promote the development and evolution of organizational capabilities only when they form a complementary coupling with adaptation mechanisms (Davenport et al., 2018; Bansal et al., 2023). (Mikalef et al. 2020) and (Yeow et al. 2018) show that technology's enabling effects are constrained by weak integration, low adaptability, and delayed structural change, hindering the “deployment–embedding–construction” pathway and underscoring the role of structural disruptions in innovation.

Technological innovation empowers organizations through a structural pathway, with its core lying in driving process optimization, organizational reconfiguration, and structural redesign, thereby activating the underlying mechanisms of capability evolution (Babu et al., 2021). Research has shown that the deployment of digital technologies can trigger process sensitivity and structural adaptation mechanisms, leading to cross-level process reengineering and the reshaping of organizational boundaries (Zeng et al., 2019; Yang et al., 2023), while simultaneously improving efficiency and innovation execution in resource allocation and cross-departmental collaboration (Wu et al., 2022). This process relies not only on internal structural disruptions but also on the integration of external technological resources, enhancing firms' collaborative responsiveness by reconfiguring data processes, platform interfaces, and collaborative networks (Li et al., 2022; Pittaway and Montazemi, 2020; Riasanow et al., 2020; Saragih et al., 2024; Troise et al., 2022; Barlette and Baillette, 2020; Belhadi et al., 2022; Zhong and Ren, 2023; Zhou and Li, 2023; Cannas, 2021; Chatfield and Reddick, 2019; Chatterjee et al., 2022; Chirumalla, 2021; Chouaibi et al., 2022; Eslami et al., 2021; Felsberger et al., 2020; Ghobakhloo et al., 2021; Jackson, 2019; Kastelli et al., 2022; Magistretti et al., 2021), together constituting the explanatory logic linking technological innovation and capability development. The enabling effect of technological innovation stems not from technology deployment itself, but from the structural transformations it induces and their integration with external resources (Kristoffersen et al., 2021).

The impact of technological innovation on organizational capabilities often depends on the support of cognitive mechanisms. (Li et al. 2021), from the perspective of alliance knowledge acquisition, demonstrate that a firm's absorptive cognitive capacity affects both the likelihood and extent to which external technological information can be embedded into business processes, thereby influencing transformation outcomes and the release of capabilities under certain conditions. (Wang et al. 2020) develop a cognitive-based explanatory model showing that organizations can strategically respond to technological change through cognitive reconstruction, which in turn guides resource allocation and the gradual evolution of capability structures. (Chen et al. 2022), in the context of industrial internet platform adoption, find that cognitive adaptation fosters process reconfiguration and practice renewal, while simultaneously strengthening resource integration and capability building. Cognitive foundations filter and translate information, shaping the transformation of technological inputs into capabilities influenced by external technologies, experience, and strategic choices (Mariani et al., 2023).

Technological innovation drives the evolution of organizational capabilities through an ecosystem-based explanatory process, in which platform structures and ecosystem collaboration serve as the core pillars (Ghobakhloo and Fathi, 2019). Firms that build open platforms and collaborate with ecosystem partners can share resources, reconfigure structures, and co-evolve capabilities, enhancing adaptability and innovation (Li et al., 2022). Platform leaders, through institutional design and role configuration, foster multilateral collaboration and value co-creation (Sun and Gregor, 2023). Ecosystem embedding then channels technological innovation into capability development, shaped by governance models, diverse actors, and sustained collaboration (Nisar et al., 2020).

Technological innovation strengthens firms' environmental governance through a sustainability pathway, which subsequently enhances organizational performance (Sultana et al., 2021). Green technological innovation, as (He et al. 2020) argue, fosters dynamic capabilities that jointly improve governance and operations, with knowledge integration and strategic sensitivity serving as key explanatory mechanisms (Gong et al., 2022). The adoption of green digital technologies further optimizes processes, reconfigures capabilities, and creates a reinforcing cycle between governance and performance (Li et al., 2022; Wang et al., 2020). Cultivating green capabilities sustains performance, shaped by resource breadth, accumulation depth, and institutional support (Zeng et al., 2019; Tian et al., 2023).

Knowledge collaboration refers to the processes of sharing, integrating, and co-creating knowledge across internal and external actors, spanning hierarchical levels, departments, and organizations (Nonaka and Takeuchi, 1997; Faraj et al., 2011). In the digitalization context, it serves as a explanatory linkage between technological enabling and capability development. Through information sharing and cognitive alignment, it enhances environmental sensing and responsiveness (Li et al., 2021). At the same time, diverse knowledge integration and collaborative decision-making enable resource optimization and capability reconfiguration (Nambisan et al., 2017; Cui et al., 2022). However cognitive divergence, structural rigidities, and weak trust often constrain cross-boundary flows, limiting the transformation of digital potential into capability building (Jarvenpaa and Majchrzak, 2016). Effective collaboration therefore depends on openness, aligned incentives, and transparent governance (Blanka et al., 2022).

The interplay between external knowledge acquisition and absorptive capacity forms a central pathway for capability evolution in digitalization. As (Li et al. 2021) show alliances embed external technologies, while absorptive capacity determines their effective transformation. Digital platforms further expand multi-source knowledge acquisition and, by strengthening absorption and transformation, enable cross-boundary integration and capability reconfiguration (Wu et al., 2022). Regional digital infrastructure also improves firms' ability to identify and integrate external knowledge, thereby reorganizing internal resources and advancing capability development (Zeng et al., 2019).

Cross-organizational and cross-domain knowledge sharing is a key mechanism for capability building in digital ecosystems. During transformation, firms rely on dynamic capabilities to develop stakeholder platforms that enable real-time integration and value co-creation (Matarazzo et al., 2021). Digital platforms also connect diverse actors, such as farmers and retailers, facilitating knowledge exchange and recombination that enhance adaptability and innovation in dynamic contexts (Pagani and Pardo, 2017). However the effectiveness of such collaboration depends on platform openness, trust, and governance mechanisms (Rachinger et al., 2019).

The construction of internal knowledge systems and the reconfiguration of capabilities form a central explanatory process through which digital transformation turns technological potential into sustainable advantage (Siachou et al., 2021). Drawing on the ROOTCLOUD case, (Li et al. 2022) show that industrial internet platforms enable supplier-side knowledge integration and cross-domain sharing, building supply chain systems that strengthen knowledge management and transformation. (Björkdahl 2020) study of 26 manufacturers demonstrates that this process requires not only new technologies but also redesigned structures and work practices to orchestrate data, capabilities, and assets for value creation and capture. (Baiyere et al. 2020) highlight the need for iterative reconfiguration within cycles of digital exploration and exploitation, while (Warner and Wäger 2019) stress the joint role of strategic renewal, capability building, and cultural shaping in leveraging dynamic capabilities under digital disruption.

### Cognition–adaptation mechanisms–organizational capabilities

Cognition refers to the processes through which individuals or organizations acquire, interpret, and construct meaning from information, encompassing environmental sensing, information processing, and judgment-based decision-making (Weick, 1995; Cohen and Levinthal, 1990). In the digitalization context, cognition has expanded from a purely human psychological activity to technology–human collaborative cognitive mechanisms, including data sensing, intelligent analytics, and organizational responsiveness (Galbraith, 1973; Zahra and George, 2002). This study conceptualizes cognition as comprising three sub-dimensions—perception, reflection, and transformation—which together explain not only how organizations recognize external changes but also how cognition connects to organizational capability evolution through adaptation mechanisms. While most studies emphasize the direct role of cognition in driving organizational capabilities or innovation performance, a smaller body of research proposes a “cognition–adaptation mechanisms–organizational capabilities” explanatory pattern, highlighting the role of structural transformation in capability development. In the following sections, we discuss how perception, reflection, and transformation, respectively, operate through adaptation mechanisms to influence organizational capabilities.

In the digitalization context, perception is regarded as a core prerequisite for organizational responses to uncertainty, functioning across three dimensions: environmental scanning, sensemaking, and signal recognition. Environmental scanning, supported by organizational data capabilities and technological infrastructure, enables the detection of external disturbance signals (Dabrowska et al., 2022). Sensemaking involves the interpretation of information and contextual reframing, serving as a foundation for resource allocation and strategic judgment (Mele et al., 2024). Signal recognition allows organizations to extract key changes from complex data and initiate response routines, with its effectiveness jointly constrained by employees' digital literacy, technological understanding, and collaborative mechanisms (Cetindamar Kozanoglu and Abedin, 2020). However, the perception process is often disrupted by limitations in knowledge structures, interpretive ambiguity, and response inertia, particularly in the early stages of digital transformation, where a disjointed chain of “seeing–recognizing–responding” is more likely to occur (Faraj et al., 2018; Leonardi, 2021). In this context, adaptation mechanisms serve as the explanatory link between perception and organizational capabilities, manifested in anticipatory adjustments, process reconfigurations, and resource reallocations in response to external disturbances (Zammuto et al., 2007). Further studies show that when perceptual capabilities are embedded within data-driven and platform-based structures, organizations are better able to achieve cross-level alignment, thereby shortening response cycles, enhancing structural flexibility, and fostering rapid experimentation (Davenport et al., 2018). Thus, perception is not merely an input stage of information processing, but also a driver of the co-generation of agility, resilience, and innovation capabilities through adaptation mechanisms (Volberda et al., 2021).

As part of cognitive mechanisms, reflection has become a crucial link between data feedback and capability reconfiguration in the digital context, helping organizations remain sensitive and adaptive under uncertainty (Li et al., 2018). This process depends not only on attentional allocation and information acquisition but also on platform-based ecosystems and knowledge systems that foster cognitive flexibility (Zeng et al., 2019; Di Vaio et al., 2021). Drawing on a case study of SMEs on Alibaba's cross-border platform, (Li et al. 2018) describe an endogenous pathway of “cognitive renewal–capability generation,” showing how reflection reshapes opportunity recognition and resource allocation, though the findings are shaped by the Chinese platform context. (Feliciano-Cestero et al. 2023) add that reflection within inter-organizational networks increases sensitivity to external dynamics and supports structural flexibility. Unlike the traditional linear “cognition–decision” model, digital reflection relies on feedback from knowledge management systems to sustain cognitive iteration (Di Vaio et al., 2021).

With the growing use of intelligent algorithms and data platforms in organizational systems, transformation has become a central pathway for restructuring capability architectures. Research distinguishes between structural and strategic mechanisms: the first involves process reorganization and role reallocation under technology adoption, while the second concerns shifts in cognitive logics and strategic trajectories (Jafari-Sadeghi et al., 2023). In practice, these mechanisms often diverge—technological deployment moves quickly, but structural and cognitive adjustments lag, producing a “technology embeddedness–organizational rigidity” paradox (Bhandari et al., 2023). (Malodia et al. 2023) and (Scuotto et al. 2022) further show that transformation succeeds only when firms display sufficient structural responsiveness and strategic adaptability to translate emerging technologies into actionable platforms and shared value orientations. In reality, however, policy-driven rapid deployments often collide with entrenched bureaucratic routines and performance-oriented logics, leaving cognitive feedback loops and institutional support underdeveloped (Madan and Ashok, 2022). For example, even after cloud migration, legacy reliance on Excel spreadsheets often persists, reflecting the structural tensions between digital deployment and organizational processes (Lanzolla et al., 2020). Such “surface-level adoption,” lacking information integration capabilities, fails to embed deeper transformation mechanisms, whereas organizations capable of path reconfiguration are more likely to achieve embedded and sustainable progress (Scuotto et al., 2022).

### Collaboration–adaptation mechanisms–organizational capabilities

In the context of digital transformation, collaboration is defined as the mutual embedding and dynamic interaction of organizational actors in resources, knowledge, and processes, whereby information sharing and capability complementarity jointly enhance the capacity to cope with complex environments (Arias-Pérez et al., 2021). Its core process lies in the dual processes of knowledge acquisition and knowledge utilization in open innovation: firms embed partners' technological, market, and operational knowledge into internal processes and integrate them with their own capabilities, thereby transforming information flows into capability building (Pagani and Pardo, 2017; Rachinger et al., 2019; Matarazzo et al., 2021). However, cross-organizational collaboration faces substantial challenges, including goal divergences among multiple actors, risks related to intellectual property and data security, as well as platform dependency and power asymmetries within digital ecosystems (Arias-Pérez et al., 2021). Representative studies reveal not only the structural mechanisms of collaboration but also its critical role in coping with market uncertainty, shortening innovation cycles, and strengthening organizational resilience.

#### Internal processes

In the context of digital transformation, internal process collaboration is defined as the dynamic optimization of organizational structures, information flows, and work practices to achieve efficient orchestration of cross-departmental resources and rapid responsiveness to environmental changes. Research indicates that digitalization relies not only on the adoption of new technologies but also on the systematic reconfiguration of organizational structures and processes to enable the efficient coordination of data, capabilities, and assets across the entire value chain (Warner and Wäger, 2019; Björkdahl, 2020; Baiyere et al., 2020). Digital infrastructures such as industrial internet platforms can empower supply chain knowledge integration and cross-departmental process optimization, thereby enhancing internal knowledge management and collaboration efficiency (Nayal et al., 2022). At the same time, open innovation requires internal processes to be capable of absorbing external knowledge and rapidly transforming it into executable solutions, which drives greater process flexibility and adaptability (Urbinati et al., 2020; Pagani and Pardo, 2017). However, the digital reconfiguration of internal processes continues to face challenges such as cross-departmental conflicts of interest, balancing process standardization with flexibility, and ensuring data security—all of which determine the effectiveness and sustainability of internal processes within the collaboration–adaptation explanatory pathway.

#### Ecosystems

Digital transformation is extending collaboration mechanisms beyond the boundaries of the firm to include customers, suppliers, and broader platform ecosystems. Through dynamic capabilities, firms integrate internal knowledge systems with the data, processes, and resources of multiple external actors into shared platforms, thereby enabling cross-boundary value co-creation and capability co-development (Matarazzo et al., 2021; Pagani and Pardo, 2017; Urbinati et al., 2020).

#### Resource flows

Digitalization is driving the evolution of strategic alliances from static contracts toward fluid collaboration, characterized by a shift from one-off transactions and fixed terms to long-term, embedded cooperation based on the continuous exchange of knowledge and resources. Within the framework of open innovation, (Arias-Pérez et al. 2021) point out that alliance relationships, through the dual processes of knowledge acquisition and utilization, become deeply embedded in firms' innovation capability building, forming adaptive mechanisms of fluid collaboration. Similarly, (Pagani and Pardo 2017) and (Urbinati et al. 2020) argue that digitalization transforms interfirm cooperation. Contract-based arrangements are replaced by cross-domain platforms for data and knowledge sharing, enabling real-time integration and joint capability development. According to (Rachinger et al. 2019), fluid collaboration allows continuous adaptation of cooperative structures and reconfiguration of business models in response to environmental change. This dynamic view contrasts with the stability assumptions underpinning traditional contract-based alliances.

### Adaptation mechanisms

Antecedent conditions such as enabling, cognition, and collaboration do shape organizational capabilities. However, in dynamic and uncertain contexts, these factors alone cannot ensure effective evolution or continuous renewal of capabilities (Deng et al., 2021). This study introduces adaptation mechanisms as an explanatory process linking digital enabling with capability building, understood as sensing external changes, assimilating new knowledge, and reconfiguring capability portfolios for adaptation and innovation (Zhang Y. et al. 2023). In this review, we use the term “mechanism” to define the underlying process logic explaining how digitalization shapes organizational capabilities. In the empirical studies reviewed, these mechanisms are often operationalized as mediators (and in some cases moderated by contextual factors), and we describe adaptation mechanisms as theoretical explanatory processes, while retaining a mechanism-based theoretical framing. Adaptation mechanisms are not a single organizational response but rather consist of complementary dimensions—including absorptive capacity, dynamic capabilities, and design-oriented transformation capabilities (Warner and Wäger, 2019). Although these mechanisms are analytically distinguished, they are closely interconnected in the process of capability development. Absorptive capacity enables organizations to recognize, assimilate, and apply external knowledge (Zahra and George, 2002), while dynamic capabilities emphasize the ability to integrate and reconfigure organizational resources in response to technological change (Teece et al., 1997). Platform capabilities further support coordination and interaction across digital infrastructures and ecosystems (Yoo et al., 2010). Rather than functioning independently, these mechanisms operate as complementary processes that jointly facilitate how organizations translate digitalization into capability development.

Together, these mechanisms determine how organizations translate enabling structures and cognitive inputs into capability evolution. In doing so, they shape the potential for differentiated advantage in complex environments.

#### Absorptive capacity

Studies in China, India, and Croatia show that absorptive capacity plays a key explanatory role in linking digital enabling and organizational capabilities. Firms use both potential and realized absorptive capacities to identify, assimilate, and transform external knowledge (Deng et al., 2021). Even under resource-constrained conditions, organizations can rely on digital technologies to integrate, translate, and reutilize knowledge to enhance adaptability (Nayal et al., 2022). Within the framework of open innovation, absorptive capacity further accelerates the speed and quality of new product development and, in complementarity with digital applications, strengthens competitive advantage (Dabić et al., 2023).

#### Dynamic capabilities

Based on cross-industry and multi-domain cases, scholars highlight that dynamic capabilities play a central explanatory role in digital transformation processes. Firms progress by sensing, seizing, and transforming, which together drive strategic renewal, capability building, and cultural shaping (Warner and Wäger, 2019). At the implementation level, organizations orchestrate cross-departmental and cross-level resources, flexibly adjust processes, structures, and value-creation models, and thereby sustain resilience and agility under environmental uncertainty (Chen and Tian, 2022).

#### Platforms

From the platform perspective of adaptation mechanisms, platform-based digital infrastructures enhance firms' ability to sense, integrate, and reconfigure resources across organizational boundaries by aggregating multi-party data resources and standardizing interfaces and service modules. In dynamic environments, this accelerates knowledge flows and capability evolution, while significantly improving the efficiency with which digital enabling is translated into innovation performance (Li et al., 2021; Zhang X. et al., 2023).

## Future directions

We contend that theorizing should shift from static chain thinking to a dynamic systems perspective to more systematically reveal how digitalization drives the evolution of organizational capabilities. This systems view reconceptualizes the digitalization–adaptation–capability nexus as boundary-spanning, multi-actor, and multi-contextual, and it motivates four avenues: (i) deepening single mechanisms, (ii) cross-mechanism and cross-level integration, (iii) contextual contingencies, and (iv) external theoretical lenses.

### Deepening single-mechanism research

Although existing studies have highlighted the importance of enabling mechanisms for capability formation—from digital infrastructure and technological innovation to cross-organizational knowledge collaboration—most treat them as static inputs and lack a systematic portrayal of how enabling elements are adapted, internalized, and transformed within dynamic adaptation processes. In particular, the contextual boundaries of their effects remain unclear across different institutional settings, resource constraints, and ecosystem complexities. This static orientation limits our understanding of how the enabling chain operates under varying conditions.

Future research could, first, focus on the dynamic evolution of enabling elements, tracing the entire process from deployment to capability embedding, and comparing differentiated pathways across governance structures, levels of external dependence, and technological configurations. Second, it should explore the interactive mechanisms among technological innovation, knowledge collaboration, organizational climate, and human characteristics, thereby uncovering their complementary or substitutive roles in capability building. Finally, scholars are encouraged to integrate enabling with cognition and collaboration mechanisms within comprehensive models, employing longitudinal tracking and cross-case comparisons to reveal their relative weights and nonlinear effects throughout the digitalization lifecycle, thus providing a more holistic understanding of the complexity of digital transformation.

Digitalization is reshaping the cognitive frameworks of managers and employees, enabling organizations to sense external signals more acutely and identify opportunities in uncertain contexts. Specifically, executives' digital mental models, employees' digital literacy, and cross-cultural information processing capabilities can accelerate the “recognition–interpretation–response” cycle, thereby driving rapid resource reconfiguration and business model adjustment, which in turn strengthen organizational dynamic capabilities and resilience (Teece, 2007; Nambisan et al., 2019). Cognitive mechanisms not only influence the depth of technology and process integration but also shape absorptive capacity and innovation speed in multinational expansion and cross-boundary collaboration. However, most existing studies remain at the level of abstract conceptualization, lacking systematic measurement of cognitive mechanisms and nuanced portrayals of their functioning across different stages of digitalization and in diverse cultural contexts.

Future research could prioritize the development of operationalized indicators of cognitive capabilities (e.g., breadth, depth, and flexibility) and employ longitudinal designs to uncover their dynamic evolution across different stages of digital transformation. Scholars should also investigate how contextual variables—such as environmental uncertainty, institutional constraints, and industry life cycles—moderate the cognition–adaptation pathway. At both the micro and organizational levels, future studies may analyze the interactive effects of executive traits, employees' digital literacy, and cross-functional cognitive integration. Finally, it is recommended that cognition be incorporated into integrated models alongside enabling and collaboration mechanisms to examine their complementary and substitutive relationships, thereby revealing the full-spectrum role of cognition within the digitalization–adaptation–capability chain.

Existing studies suggest that digitalization, by fostering collaborative networks across departments, organizations, and even ecosystems, significantly enhances the efficiency of information flows and resource reconfiguration, thereby strengthening organizations' responsiveness and adaptability under environmental uncertainty. Platformization and ecosystem participation provide the structural foundation for multi-party collaboration, while data sharing and standardized interfaces accelerate joint innovation and market entry. At the same time, trust and governance mechanisms play a critical explanatory role in transforming collaboration into organizational capabilities. However, most research has concentrated on technological or operational dimensions, with relatively limited attention to the quantification of collaboration quality, the examination of its functioning across levels and cultural contexts, and the identification of potential negative effects (Adner, 2017; Jacobides et al., 2018).

Given that much of the existing research remains focused on whether collaboration exists rather than on its quality, future studies should prioritize the development of measurement systems for collaboration quality and examine the underlying mechanisms through which it translates into specific capability dimensions. In particular, in multi-market, multi-institutional, and highly digitalized industries (e.g., finance and insurance), further analysis is needed to understand how external embeddedness interacts with organizational agility to enhance adaptation speed. Multi-level and multi-actor analyses can also help reveal the boundary conditions of risks such as resource lock-in and path dependence within ecosystems. By combining longitudinal tracking with cross-context comparisons, scholars may further depict the bidirectional flow of knowledge between global platforms and local markets, and explain how such flows shape organizational capabilities under varying competitive and institutional environments. Through these efforts, future research will contribute to a more comprehensive understanding of the mechanisms of collaboration in complex digital environments (Arias-Pérez et al., 2021).

In the review of the three explanatory patterns of enabling, cognition and collaboration, we have revealed the diverse paths through which digitalization promotes the formation of organizational capabilities through different mechanisms. However, the linear segmentation of existing research still fails to fully explain the complex logic of digitalization in different contexts and among multiple subjects. To break through this limitation, we advocate regarding “digitalization—adaptation mechanism—organizational capability” as a dynamic multi-path system rather than a fragmented single chain. This system not only reflects the complementary, substitutive and interactive effects among mechanisms, but also needs to incorporate the integration logic of “multi-actor” and “multi-context”, thereby constructing a comprehensive framework that spans boundaries. To more systematically reveal how digitalization drives the evolution of organizational capabilities, we contend that theorizing should shift from static chain thinking to a dynamic system perspective that reconceptualizes digitalization–adaptation–capability linkages as boundary-spanning, multi-actor, and multi-context processes.

To make this shift concrete, we advance the Digital Capability System (DCS) as a named theoretical lens. DCS departs from linear, one-way chains by conceptualizing digitalization-driven capability evolution as a recursive system of interdependent mechanisms. First, DCS highlights dynamic feedback loops: adaptation mechanisms not only explain the effects of enabling, cognition, and collaboration but also reshape their subsequent efficacy over time, generating path dependence and potential lock-in as organizations learn and reconfigure (Eisenhardt and Martin, 2000; Teece, 2007). Second, DCS emphasizes cross-level integration: micro-level cognitions and behaviors aggregate to meso-level routines and platform practices, which in turn structure ecosystem-level coordination and capability reconfiguration, addressing conflations noted in prior work (Warner and Wäger, 2019; Björkdahl, 2020). Third, DCS foregrounds contextual contingencies: institutional settings, industry digital intensity, and resource constraints systematically moderate how mechanisms interact (Adner, 2017; Jacobides et al., 2018). This system perspective resonates with complexity and system-dynamics thinking (Sterman, 2000) and provides a unifying template for comparative, longitudinal, and multi-actor designs.

Implications for research design follow directly. Longitudinal panels and process tracing can identify recursive feedback; cross-country and cross-industry comparisons can test contextual contingencies; and system-dynamics or agent-based simulations can probe non-linear complementarities among mechanisms. Importantly, DCS treats enabling, cognition, collaboration, and adaptation as a constellation whose weights vary across phases of the digitalization lifecycle, inviting studies that estimate mechanism relative importance and threshold effects under heterogeneous governance structures and platform architectures. In short, DCS transforms the digitalization–adaptation–capability nexus from fragmented linear pathways into a holistic, recursive system, thereby deepening the theoretical foundations for explaining capability evolution in digitally intensive and boundary-spanning contexts.

### Cross-mechanism integration research

Although enabling mechanisms such as digital infrastructure, technological innovation, and knowledge collaboration greatly enhance the accessibility of information and resources, whether they can truly be transformed into organizational capabilities depends on the management's ability to perceive, interpret, and reflect on technological potential. High levels of digital infrastructure, if lacking cognitive drivers, may remain “available” rather than “usable.” Conversely, when executives possess cross-domain knowledge, data interpretation skills, and strategic sensing capabilities, enabling mechanisms are more likely to integrate external knowledge through absorptive capacity and achieve rapid reconfiguration with the support of dynamic capabilities. For example, future research could examine whether managerial cognitive differences, under the same level of digitalization, significantly influence the speed and quality with which enabling mechanisms reshape business models and adjust value propositions, and employ longitudinal tracking to uncover the critical thresholds and nonlinear effects of cognition within the technology–adaptation chain.

Cross-departmental, cross-market, and cross-border collaboration mechanisms broaden organizations' sources of information and resource mobilization, but information alone does not automatically generate adaptive advantages. Cognitive mechanisms play a critical role of “interpretation–integration–transformation,” converting multi-source information into executable strategic initiatives. Future research could systematically analyze how different types of collaboration (e.g., internal process integration vs. ecosystem network cooperation) shape cognitive boundaries and, through platform capabilities and dynamic capabilities, translate cognitive outcomes into collaborative actions. Moreover, in cross-cultural or cross-institutional contexts, collaboration mechanisms may create cognitive tensions and interpretive biases, offering a novel entry point for exploring the boundary conditions under which conflict and synergy coexist in the cognition–collaboration interaction.

Enabling mechanisms provide data, communication, and process support for collaborative relationships, while frequent interactions and knowledge exchanges within collaboration, in turn, drive the optimization and iteration of enabling tools and platform architectures. Future research could explore how this bidirectional relationship generates sustained capability accumulation effects through absorptive capacity. For instance, when cross-functional teams share real-time data platforms, they not only improve task execution efficiency but may also, through feedback mechanisms, prompt platform functionalities to become better aligned with business needs, thereby forming a cyclical pathway of “collaboration-driven enabling optimization—enabling -enhanced collaboration performance.

When enabling, collaboration, and cognition mechanisms operate in synergy, they can generate a significant capability acceleration effect: enabling drives the smooth flow of information, collaboration expands the breadth of information coverage, and cognition optimizes the filtering and utilization of information while simultaneously guiding the refinement of enabling and collaboration. In this process, adaptation mechanisms play a pivotal role, which may be led by absorptive capacity (rapid integration of new knowledge), dynamic capabilities (reconfiguration of processes and resources), or platform capabilities (enabling efficient multi-party interaction), depending on the context. Future research could develop multi-level integrative models to examine how different dominant combinations of mechanisms vary in their effects on capability generation efficiency, innovation speed, and resilience building, thereby providing a more systematic framework for understanding the evolution of organizational capabilities in the digital context. While the majority of studies reviewed report positive effects of digitalization on organizational capabilities, several studies also highlight mixed or context-dependent outcomes. These findings suggest that the influence of digitalization may vary depending on organizational conditions, implementation processes, and technological maturity. Moreover, the existing literature may be subject to potential publication bias, as studies reporting positive relationships between digitalization and capability development are more likely to be published than those reporting null or negative results.

### Cross-actor and cross-context extensions

Existing studies generally treat digitalization mechanisms as universally effective explanatory patterns within organizations, however the heterogeneity across actors and contexts has rarely been systematically examined, which limits the external validity and explanatory power of current findings. In fact, differences across industries, regions, and actors often lead to the same mechanism chain producing divergent, even contradictory outcomes. In high digital-density industries such as finance and ICT, digitalization is often a survival necessity, accelerating cognitive restructuring and dynamic capability iteration. By contrast, in low digital-density industries such as traditional manufacturing or public services, digitalization tends to manifest as an external policy or institutional pressure, often accompanied by resource slack and capability misalignment. This divergence raises a critical debate: is digitalization primarily an endogenous driver of self-reinforcing capability development, or an externally imposed institutional constraint?

Similar contradictions also appear across institutional environments. In highly regulated European markets with strict compliance requirements, digital enablement mechanisms are often constrained by institutional pressures, and their positive effects may be weakened or even reversed. In relatively loose institutional environments such as emerging markets, however, firms are often able to leverage flexible collaboration and rapid trial-and-error to trigger stronger capability reconfiguration. This contrast raises a fundamental question: why does the same digital investment yield entirely opposite outcomes across different regions? Such comparisons highlight the urgent need for research to examine how institutional strength, market maturity, and cultural variation shape the boundary conditions of digitalization mechanisms.

Differences across actors should not be overlooked either. Most existing studies focus on the strategic cognition of top management, but in practice, frontline employees' digital literacy, incentive structures, and routines often determine whether capability transformation succeeds. At the same time, the extent of external partners' involvement in ecosystems significantly affects the stability and sustainability of collaboration mechanisms. Thus, future research needs to move beyond the single pathway of “top management cognition—strategic orientation” and systematically examine the interactions among multiple actors. This includes exploring whether tensions between strategic logic and operational routines, or between internal goals and external partners' demands, may lead to ruptures in the digitalization–capability chain. Methodologically, cross-industry matched studies, multi-case comparisons, natural experiments, and multilevel modeling can be employed to uncover heterogeneity across industries, institutional environments, and actors, thereby advancing our understanding of the boundary conditions of digitalization.

### Introducing external theories to enrich the field

Although the digitalization–adaptation mechanism–organizational capability process reveals the core explanatory logic, existing studies remain insufficient in explaining the divergence and contradictions in its effects. Future research should actively draw on external theories to enrich and expand theoretical development in this domain. The contingency perspective and contingency theory provide a valuable entry point. Current literature often assumes that digitalization mechanisms are universally valid across contexts, but differences in institutional environments, industry life cycles, and environmental uncertainty can significantly alter the direction and magnitude of their effects. Contingency theory can explain why the same enabling or collaborative pathway produces positive effects in one institutional setting but fails in another, thereby correcting the “one-size-fits-all” assumption pervasive in current research.

From a performance orientation view, digitalization can cut both ways. Result-oriented goals raise short-term efficiency but limit exploration, while learning-oriented goals build resilience by using slack resources and flexible networks to unlock digitalization's long-term potential. Goal-setting and motivation theories clarify why digitalization produces both positive and negative effects. They also offer a useful basis for future research. Empirically, researchers can track firms over time or run experiments that manipulate performance goals to test their moderating role in the digitalization–capability link.

Beyond these moderating factors, external mechanisms also matter. Resilience explains how firms absorb crisis shocks and recover over time. Slack resources show why digitalization may follow different paths under abundance vs. constraint. Organizational learning highlights whether digital practices become part of routines or remain superficial adoption. Methodologically, researchers can test these ideas through event studies, cross-context comparative experiments, or longitudinal panel data. By incorporating external theories and providing empirical identification strategies, digitalization research can not only address existing theoretical gaps but also engage in deeper dialogue with broader strategic management and organizational capability literatures.

Based on the Digital Capability System (DCS) framework, future research may empirically test the following propositions.

Proposition 1. Enabling mechanisms positively influence organizational capabilities by facilitating digital infrastructure integration and data accessibility.

Proposition 2. Cognitive mechanisms enhance organizational capabilities by improving organizational learning and decision-making quality.

Proposition 3. Collaborative mechanisms strengthen organizational capabilities by increasing cross-functional coordination and knowledge exchange.

Proposition 4. Adaptation mechanisms explain the relationship between digitalization and organizational capability development through absorptive capacity and dynamic capabilities.

Possible indicators: absorptive capacity, dynamic capability measures, innovation speed.

## Conclusion

This review synthesizes research on the digitalization–adaptation–capability nexus, identifies three core explanatory patterns—enabling, cognition, and collaboration—and clarifies the explanatory role of adaptation mechanisms. Building on this synthesis, the unified framework specifies complementarities and substitutions across mechanisms and delineates cross-actor and cross-context extensions. Together, the framework and agenda provide a coherent basis for cumulative theorizing and for empirical tests across industries and institutional settings.

## Data Availability

The original contributions presented in the study are included in the article/Supplementary material, further inquiries can be directed to the corresponding author.

## Appendix

**Table A1:** Search strategy.

Component	Search terms
Digitalization-related terms	“digitalization” OR “digital governance” OR “digital infrastructure” OR “digital transformation” OR “platform governance” OR “data governance” OR “algorithmic governance”
Organizational capability-related terms	“organizational capability” OR “dynamic capabilities” OR “absorptive capacity” OR “organizational learning” OR “resource integration” OR “innovation capability” OR exploration OR exploitation
Boolean logic	(Digitalization-related terms) AND (Organizational capability-related terms)
Web of Science query format	TS = (“digitalization” OR “digital governance” OR “digital infrastructure” OR “digital transformation” OR “platform governance” OR “data governance” OR “algorithmic governance”) AND TS = (“organizational capability” OR “dynamic capabilities” OR “absorptive capacity” OR “organizational learning” OR “resource integration” OR “innovation capability” OR exploration OR exploitation)
Search fields	Topic search (TS): title, abstract, author keywords, keywords plus
Database	Web of science core collection
Time span	1949–2025
